# Early intervention for subthreshold panic disorder in the Netherlands: A model-based economic evaluation from a societal perspective

**DOI:** 10.1371/journal.pone.0193338

**Published:** 2018-02-21

**Authors:** Robbin H. Ophuis, Joran Lokkerbol, Mickaël Hiligsmann, Silvia M. A. A. Evers

**Affiliations:** 1 Department of Public Health, Erasmus University Medical Center, Rotterdam, The Netherlands; 2 Centre of Economic Evaluation, Trimbos Institute (Netherlands Institute for Mental Health and Addiction), Utrecht, The Netherlands; 3 Rob Giel Research Center, University Medical Center Groningen, Groningen, The Netherlands; 4 Department of Health Services Research, CAPHRI Care and Public Health Research Institute, Maastricht University, Maastricht, The Netherlands; Deakin University, AUSTRALIA

## Abstract

**Background:**

Panic disorder (PD) is associated with impaired functioning and reduced quality of life. In the Netherlands, almost 2% of the population experiences clinically relevant panic symptoms without meeting the diagnostic criteria for PD, which is referred to as subthreshold PD (STHPD). Evidence suggests that subthreshold mental disorders may have a similar impact on quality of life and functioning in comparison with full-blown mental disorders, which draws attention to the need for interventions for STHPD. These interventions are currently not systematically provided in clinical practice. This study aims to investigate the population cost-effectiveness of adding a CBT-based early intervention for adults with STHPD to the existing health care for people with PD in the Netherlands.

**Methods:**

A health-economic Markov model was constructed in order to compare quality adjusted life-years (QALYs) and societal costs of adding an early intervention to usual care for PD. The model compares usual care with an alternative program in which usual care is supplemented with a CBT-based early intervention. Input parameters for the model were derived from national sources and published literature where possible, and based on expert opinion otherwise. Probabilistic and deterministic sensitivity analyses were conducted to evaluate the uncertainty of the model input parameters.

**Results:**

On average, the added CBT-based early intervention was dominant in comparison with usual care, meaning that the early intervention yielded more QALYs at lower costs. At a willingness-to-pay threshold of €20,000 per QALY, the cost-effectiveness probability of the added early intervention was 98%. Sensitivity analyses showed that the results were robust.

**Conclusions:**

This study showed that offering an early intervention in addition to usual care for PD is potentially cost-effective, but it should be further investigated to what extent trial results can be extrapolated to the level of the population before such interventions are implemented on a large scale.

## Introduction

Panic disorder (PD) is a disabling anxiety disorder that is associated with impaired functioning and reduced quality of life [1, 2]. Individuals with PD experience unexpected panic attacks characterized by symptoms such as hyperventilation, palpitations and derealisation [3]. Important characteristics of PD are the avoidance of potential anxiety-provoking situations and the anticipated fear of having future panic attacks [3].

PD is associated with medically unexplained symptoms and high healthcare utilization [4, 5]. On average, individuals who experience panic symptoms visit general practitioners, medical specialists, and emergency departments more often than the general population [6].

About 2% of the Dutch population experiences clinically relevant panic symptoms without meeting the diagnostic criteria of a full-blown PD diagnosis [7]. This subclinical form of PD is generally referred to as subthreshold PD (STHPD) [8]. In the Netherlands, STHPD is almost as prevalent as full-blown PD (1.9% versus 2.2%) [7].

As a result of the currently applied diagnostic thresholds for PD, many individuals with clinically relevant symptoms might not receive appropriate treatment [9], which could lead to progression of morbidity and deterioration of quality of life resulting in full-blown PD [10, 11]. Studies have shown that subthreshold mental disorders may have a similar impact on quality of life and functioning on the level of the population in comparison with full-blown mental disorders due to a higher prevalence of subthreshold disorders [9, 11]. Although the STHPD prevalence in the Netherlands is lower than the prevalence of PD, the health impact and costs of STHPD are substantial.

The societal per capita costs for PD and STHPD in the Netherlands are respectively €8,000–10,000 and €6,000 per year [7, 12]. The indirect non-medical costs for PD and STHPD are both higher than the indirect non-medical costs for phobias, social anxiety disorder, and generalized anxiety disorder [12], which shows that that PD and STHPD are both associated with substantial productivity losses. The relatively high prevalence rate and societal costs of STHPD emphasize that there is a treatment need.

Various psychological and pharmacological interventions for PD have been shown to be effective and cost-effective [13–15], but the evidence on the effectiveness and cost-effectiveness of interventions for treating STHPD is less substantial. Meulenbeek et al. [16] showed that a cognitive behavioral therapy (CBT) based early intervention for adults with STHPD significantly reduced panic symptoms. This intervention called ‘Don’t Panic’ is group-based. An economic evaluation of this intervention resulted in an incremental cost-effectiveness ratio (ICER) of €6,000 per PD-free survival gained when compared to usual care [17].

Although these studies [16, 17] show promising results in terms of effectiveness, early interventions for treating STHPD are currently not systematically offered in Dutch clinical practice. Before such interventions can be implemented in clinical practice, further evidence on the cost-effectiveness at the population level is essential for policy makers. A health-economic model can contribute to such evidence. Therefore, this study aims to investigate the population cost-effectiveness of adding a CBT-based early intervention for adults with STHPD to the existing health care for people with PD in the Netherlands by means of a health-economic Markov model.

## Methods

The methods and reporting of this model-based economic evaluation are in concordance with the Consolidated Health Economic Evaluation Reporting Standards (CHEERS) statement and ISPOR recommendations [18, 19]. A completed CHEERS checklist has been added to the appendix (S1 Table).

### Study population and epidemiology

The study population in the model comprised the Dutch adult population aged 18–65 years [20, 21]. Prevalence and incidence estimates of PD and STHPD were obtained from the Netherlands Mental Health Survey and Incidence Study, a cohort study on the epidemiology of mental disorders in the Dutch general population [22]. The annual prevalence rates of PD and STHPD were respectively 2.2% (95%CI 1.9–2.6) and 1.9% (95%CI 1.6–2.2) [9]. DSM-III-R diagnostic criteria for PD were applied. STHPD was defined as having at least one experience of panic during the last year in a situation in which the panic attack is not considered normal [9]. This experience must have been accompanied by at least four of the thirteen panic symptoms according to the DSM-III-R, and may not be of organic cause.

The annual incidence of PD in the Netherlands was 0.52% (95%CI 0.37–0.73) [23]. Within two years, 43.3% of the PD patients and 23.8% of the STHPD patients had chronic panic complaints, whereas respectively 21.4% and 37% of the STHPD patients who reached remission had a recurrence [24].

### Usual care

The usual care scenario (Table 1) reflects usual care for PD in the Netherlands. We assumed that the recommended interventions as described in Dutch multidisciplinary treatment guideline for anxiety disorders [25] represent current clinical practice.

**Table 1:** Base-case and alternative scenario.

Intervention	Uptake usual care (%)	Uptake additional CBT-based early intervention (%)	Adherence (%)
**PD**			
CBT	8.8	8.8	70
SSRI	9.2	9.2	57
TCA	2.4	2.4	49
Combination therapy	9.0	9.0	70
**STHPD**			
CBT-based early intervention	0	10	70

Recommended psychological interventions for PD are therapist-led interventions based on cognitive or behavioral therapy [25]. In Dutch clinical practice, these interventions are generally offered combined as CBT. Therefore, CBT was considered as psychological intervention in the model. We defined CBT as a psychological intervention based on learning theory and cognitive restructuring [26].

Selective serotonin inhibitors (SSRIs) are first-choice pharmacotherapy for PD and tricyclic antidepressants (TCAs) are second-choice pharmacotherapy [25]. Both SSRIs and TCAs were added to the model as pharmacological interventions. SSRIs are first-choice pharmacotherapy as they are generally better tolerated and cause fewer adverse events in comparison with TCAs [25].

Furthermore, combination therapy consisting of pharmacological treatment and CBT was added to the model. Benzodiazepines were not considered because they are only recommended for acute and situation-specific treatment [25].

### Additional CBT-based early intervention

In order to investigate the additional effect of adding an early intervention for STHPD to the current clinical practice for PD in the Netherlands, usual care was supplemented with an early intervention for treating STHPD (Table 1).

The early intervention in the model was based on the ‘Don’t Panic’ intervention described by Meulenbeek et al. [16] and Smit et al. [17]. The early intervention is a CBT-based group course for adults aged eighteen years and older (6–12 participants) with STHPD. The group course consists of eight weekly sessions of two hours guided by a prevention worker and a mental health clinician. The group course covers psycho-education, lifestyle advice, stress coping, cognitive restructuring, in vivo exposure, and a relapse prevention training.

### Intervention uptake and adherence

In the Netherlands, 29.4% of the PD patients utilize mental health care [27]. As it is not known how often specific interventions for PD are offered, we used Australian data regarding mental health care utilization as an approximation [28]. Based on expert opinion, respectively 85% and 15% of the Dutch PD patients receiving pharmacotherapy use SSRIs and TCAs, of which one third receives combination therapy (both pharmacotherapy and CBT) due to unsuccessful treatment outcomes. Because early interventions are currently not offered systematically in the Netherlands, the uptake in usual care is set at 0%. In the scenario with the added CBT-based early intervention, the uptake rate for the early intervention is set at 10%. For the remaining interventions, we applied equal uptake rates.

We applied an adherence rate of 70% for all interventions considered in the model, assuming that the effectiveness of interventions in clinical practice is generally lower than in clinical trials. Additionally, we applied drop-out rates for SSRI and TCA treatment (respectively 18% and 30%) based on the Dutch multidisciplinary guideline for anxiety disorders [25], resulting in lower adherence rates. We conservatively assumed that non-adherent patients had no utility gain (no effect of treatment). Full intervention costs were incurred for non-adherent patients. Table 1 describes the uptake and adherence rates for usual care and the early intervention scenario.

### Structural assumptions

**Fig 1:**
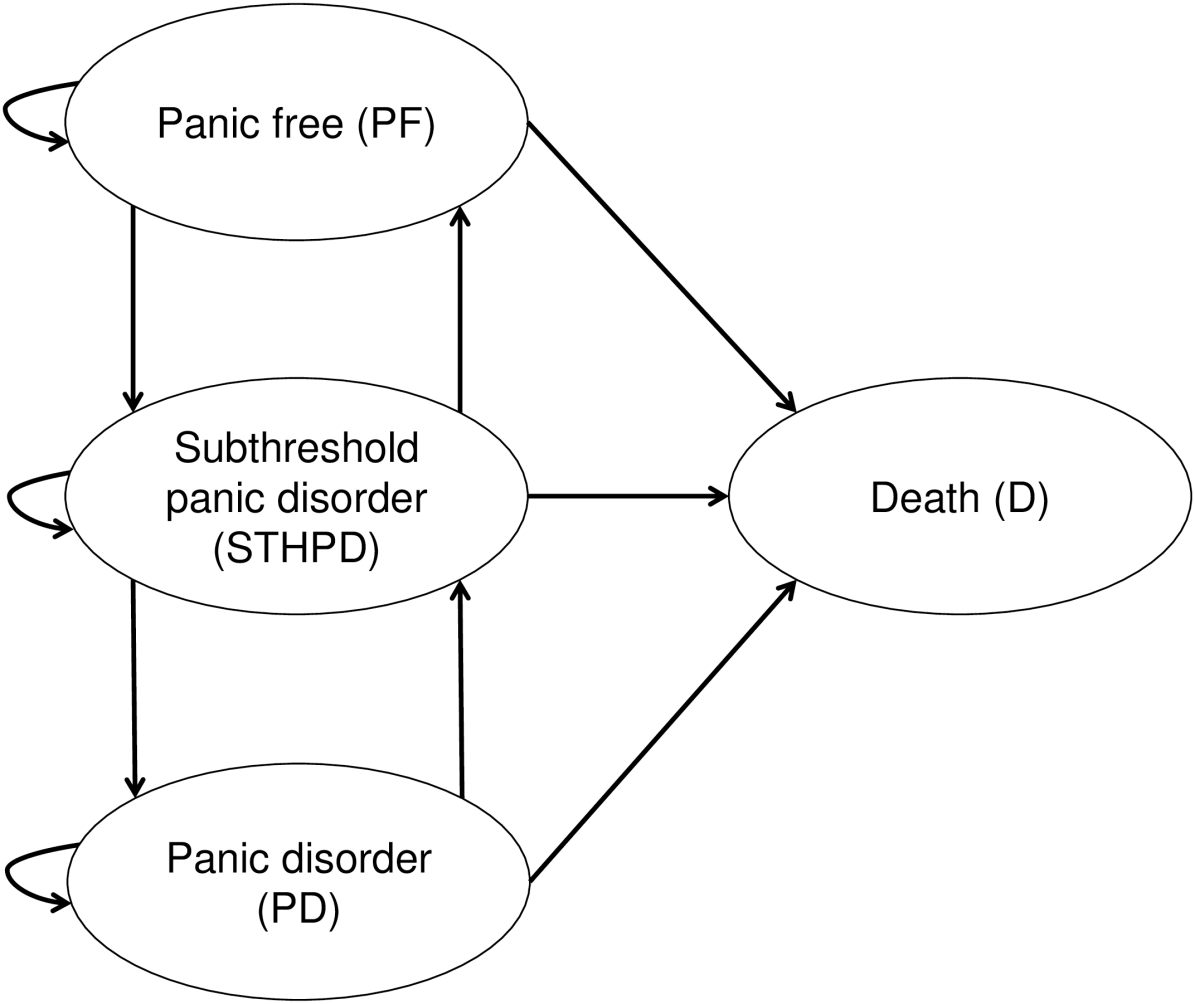
Markov model structure.

A four-state Markov model was constructed using Microsoft Excel 2013 in order to assess the incremental costs and quality adjusted life-years (QALYs) of the added CBT-based early intervention versus usual care. The model structure, as depicted in Fig 1, is based on the availability of epidemiological and clinical evidence. The cycle length was one year, corresponding to the available epidemiological evidence, which generally reports annual transition rates. Subjects either remain in the same health state or switch to a connecting health state, assuming that only one switch per cycle is possible. We applied a time horizon of five years.

Four mutually exclusive health states were considered in the model; panic-free (PF), STHPD, PD, and death. Individuals who neither met the earlier mentioned diagnostic criteria for PD and STHPD were in the PF health state. We assumed that STHPD was intermediate between PF and PD, meaning that individuals with PD can only reach the PF state by crossing the STHPD health state and vice versa. This assumption implies that the incidence of PD solely occurs from STHPD.

### Model parameters

#### Health state utilities

The effectiveness of the interventions was expressed in QALYs, which required the use of utility scores of the included health states. A utility score reflects health-related quality of life ranging from 0 (death) to 1 (full health). Utility values can be multiplied by their duration in years to calculate QALYs. One QALY gained reflects one extra life-year in full health gained. For the individuals who were not diagnosed with (STH)PD, we used a published utility score of the Dutch general population [29]. Utility data for the PD health state were derived from the European Study of the Epidemiology of Mental Disorders [30]. The utility score for the STHPD health state was derived from a Dutch disability weight study [31]. All utility scores were based on the EQ-5D. STHPD and PD utilities were multiplied with the general population utility in order to ensure that improvements from STHPD and PD to PF reflected utility gains as expected by burden of disease studies [30, 31]. This adjustment was deemed necessary to improve the comparability of health state utility values taken from different sources. In a sensitivity analysis, we investigate the impact of this correction. The health state utilities are reported in Table 2, alongside with the other parameter values.

**Table 2:** Model input parameters.

Input parameter	Deterministic value	Probabilistic distribution	Source
Discount rate costs	4%	Fixed	Dutch Guideline for Health Economic Evaluations [32]
Discount rate outcomes	1.5%	Fixed	Dutch Guideline for Health Economic Evaluations [32]
**Indirect non-medical costs per health state (annual)**			
PF	€6,643	Gamma	Based on data by Batelaan et al. and Smit et al. [7, 12]
STHPD	€9,741	Gamma	Based on data by Batelaan et al. [7]
PD	€16,771	Gamma	Based on data by Batelaan et al. [7]
**Direct medical costs (annual)**			
PF	€340	Gamma	Based on data by Batelaan et al. [7]
STHPD	€1,503	Gamma	Based on data by Batelaan et al. [7]
PD	€666	Gamma	Based on data by Batelaan et al. [7]
**Direct non-medical costs (annual)**			
PF	€99	Gamma	Based on data by Batelaan et al. [7]
STHPD	€494	Gamma	Based on data by Batelaan et al. [7]
PD	€858	Gamma	Based on data by Batelaan et al. [7]
**Health state utilities**			
PF	0.869	Normal, SE: 0.0054	[29]
STHPD	0.730	Normal, SE: 0.037	Based on Versteegh et al. [29] and Stouthard et al. [31]
PD	0.660	Normal, SE: 0.030	Based on Versteegh et al. [29] and Kaltenthaler et al. [30]
**Transition probabilities**			
PF to STHPD	0.0016	Fixed	Calculations based on epidemiology [7, 24]
STHPD to PF	0.0815	Fixed	Calculations based on epidemiology [7, 24]
STHPD to PD	0.6037	Fixed	Calculations based on epidemiology [7, 24]
*STHPD to PD transition is lowered by the early intervention*, *based on a RR of 0*.*538* [16]			
PD to STHPD	0.5214	Fixed	Calculations based on epidemiology [7, 24]
Any state to ‘Death’	0.0028	Fixed	Based on Dutch national statistics, averaged for the population aged 18–65 years [33]
**Utility gain after intervention**			
CBT	0.0327	Normal	Based on Sanderson et al. [34] and Bandelow et al. [14]
SSRI	0.0450	Normal	Based on Sanderson et al. [34] and Bandelow et al. [14]
TCA	0.0445	Normal	Based on Sanderson et al. [34] and Bandelow et al. [14]
Combination therapy	0.0568	Normal	Based on Sanderson et al. [34] and Bandelow et al. [14]
Early intervention	0.0655	Normal	Based on Sanderson et al. [34] and Meulenbeek et al.[16]
**Intervention costs (per person)**			
CBT^a^	€1,176	Gamma	[32], Expert opinion
SSRI^b^	€1,128	Gamma	[32], Expert opinion
TCA^c^	€1,200	Gamma	[32], Expert opinion
Combination therapy^d^	€2,315	Gamma	[32], Expert opinion
Early intervention^e^	€905	Gamma	[17]

^a^12 sessions in Basic mental health care.

^b^Defined Daily Dose for one year (average SSRI costs) plus 9.5 sessions (min. 8 and max. 12) in specialized mental health care.

^c^Defined Daily Dose for one year (average TCA costs) plus 9.5 session in specialized mental health care.

^d^12 general mental health institution sessions, Defined Daily Dose for one year (average TCA/SSRI costs) plus 9.5 sessions in specialized mental health care.

^e^Based on Smit et al. [17] (explained in the Methods section).

#### Health gains

Interventions aim to increase utility and thereby QALYs. In the model, QALY gains due to interventions aimed at treating PD were based on effect sizes from a recent meta-analysis on interventions for anxiety disorders [14]. The effect size of the early intervention was based on trial data [16]. Effect sizes were converted to utility gains by means of a conversion method described by Sanderson et al [34]. The effect sizes and conversion values are presented in the appendix (S2 Table). The preventive effect of the early intervention on progression from STHPD to PD, expressed as a decrease in the transition rate from SHPD to PD, was based on the study by Meulenbeek et al. [16].

#### Transition probabilities

Transition probabilities in terms of incidence, recurrence, and chronicity as described earlier reflect usual care since they were based on cohort studies in which participants had no restrictions in terms of healthcare resources. Ideally, intervention specific transition probabilities are included in the model. We used intervention specific probabilities for the early intervention, but not for the remaining interventions due to a limited availability of evidence.

#### Resource use and cost data

Direct medical costs, direct non-medical costs, and indirect non-medical costs for the PD and STHPD health states were derived from a cost-of-illness study by Batelaan et al [7]. The authors were contacted for a detailed overview of the cost categories. Direct medical costs relate directly to medical care, such as doctor visits. Direct non-medical costs are out-of-pocket costs, for example travel costs due to doctor visits. Indirect non-medical costs consist of costs due to productivity losses. We calculated the societal costs by summing up the direct medical costs, direct non-medical costs, and indirect non-medical costs. All costs are expressed in euros (€) for the reference-year 2014. We used the Dutch price index (Central Bureau of Statistics, the Netherlands) in order to express costs in 2014 euros.

#### Intervention specific costs

Unit cost prices were obtained from the Dutch Guideline for Health Economic Evaluations [32]. Costs for medication were based on Dutch reimbursement rates [35]. The intervention-specific resource use is described in Table 2.

The costs for the early intervention were derived from the economic evaluation of the ‘Don’t panic’ intervention by Smit et al. [17]. The intervention costs were €905 per patient (indexed for the year 2014). The intervention consists of eight CBT sessions of two hours each by a prevention worker and a clinician, followed by one booster session three months after completion.

#### Cost-effectiveness analysis

Mean costs and QALYs were estimated for usual care and the added CBT-based early intervention. Both costs and effects were discounted annually. The incremental cost-effectiveness ratio (ICER) was calculated by dividing the difference in costs between both scenarios by the difference in QALYs. The ICER represents the cost of an additional life-year in full health gained. A cost-effectiveness acceptability curve was plotted, which shows the probability that the added early intervention is cost-effective for different willingness-to-pay (WTP) thresholds. The early intervention was deemed cost-effective when the ICER is below the WTP-threshold of €20,000 per QALY, a threshold for disorders with disability weights between 0.1 and 0.4 according to the Dutch Healthcare Institute recommendations [36].

#### Handling uncertainty

A probabilistic sensitivity analysis was performed in order to take into account the uncertainty surrounding the point estimates of the input parameters. Probability distributions were assigned to the parameters, after which 10,000 iterations were performed by drawing random values for the input parameters. Utility parameters were assigned a normal distribution, and cost parameters were assigned a gamma-distribution as presented in Table 2. The mean incremental costs and QALYs were calculated by averaging the 10,000 iterations.

Deterministic sensitivity analyses explored the impact of making several assumptions: applying different uptake rates for the interventions, varying the effectiveness of the early intervention, the use of alternative time horizons of one and ten years, and the use of utility values that were not corrected for the baseline level of the general population.

## Results

Over a period of five years, usual care resulted in 3.28 QALYs (95% CI 3.01–3.48) and €59,634 (95% CI 45,097–76,084) per patient on average, whereas the added CBT-based early intervention resulted in 3.30 QALYs (95% CI 3.10–3.49) and €59,355 (95% CI 44,984–75,621) per patient. All patients with STHPD and PD were included, regardless of whether they received any intervention. When only the STHPD patients are considered, usual care resulted in 3.47 QALYs (95%CI 3.16–3.77) and €43,099 (95%CI 28,402–60,779) on average per STHPD patient, and the added early intervention 3.58 QALYs (95%CI 3.27–3.89) and €44,512 (95%CI 29,448–62,668).

**Fig 2:**
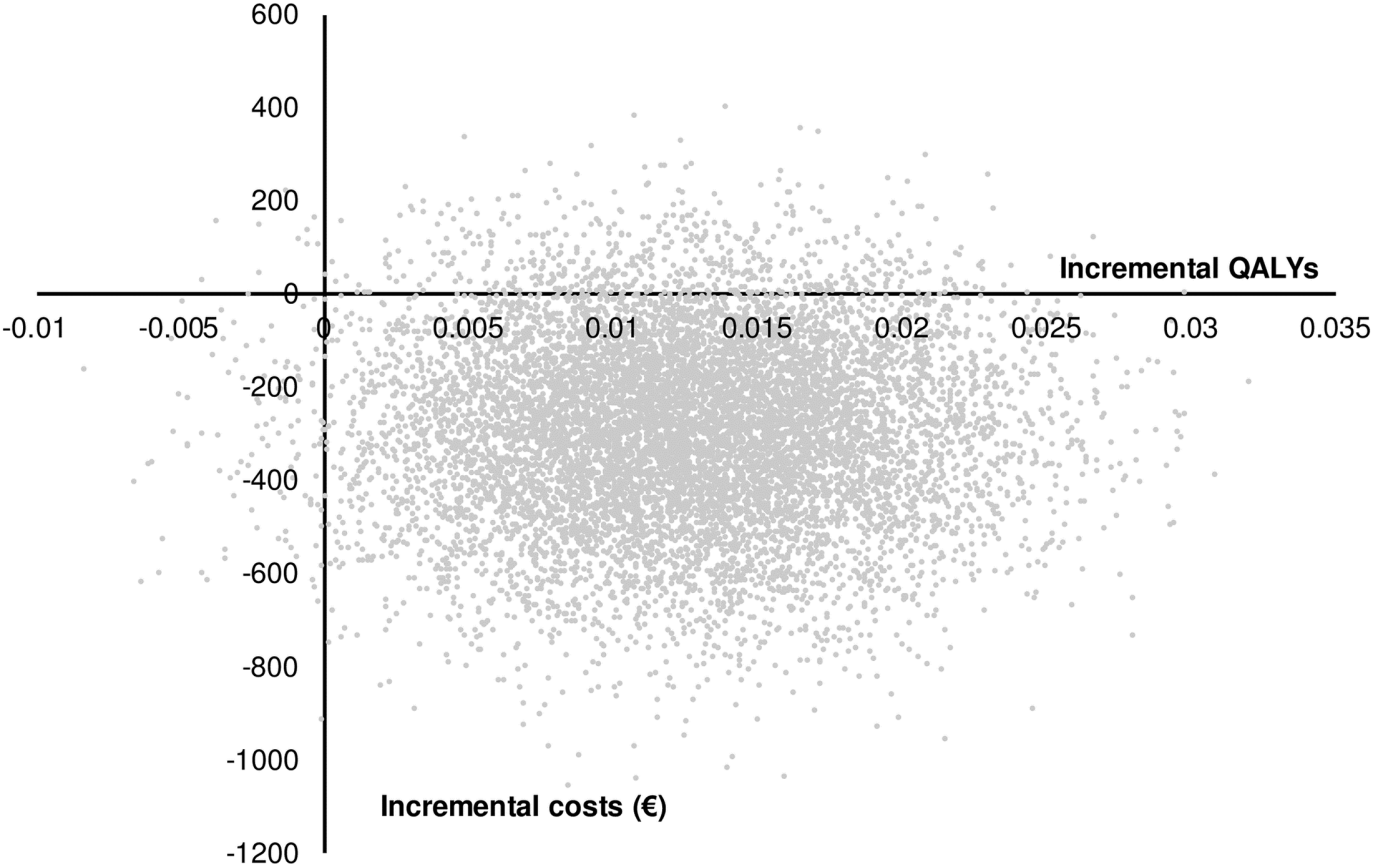
Incremental cost-effectiveness plane of the additional CBT-based early intervention versus usual care.

When both STHPD and PD patients are considered, the ICER equaled €-23,127 (95% CI -395,731–3,682) per QALY gained, meaning that the added early intervention resulted in more QALYs at lower costs on average in comparison with usual care. Fig 2 presents the cost-effectiveness plane, in which the ICERs for the 10,000 iterations are depicted. The majority of the ICERs were located in the lower-right quadrant of the incremental cost-effectiveness plane, indicating dominance.

**Fig 3:**
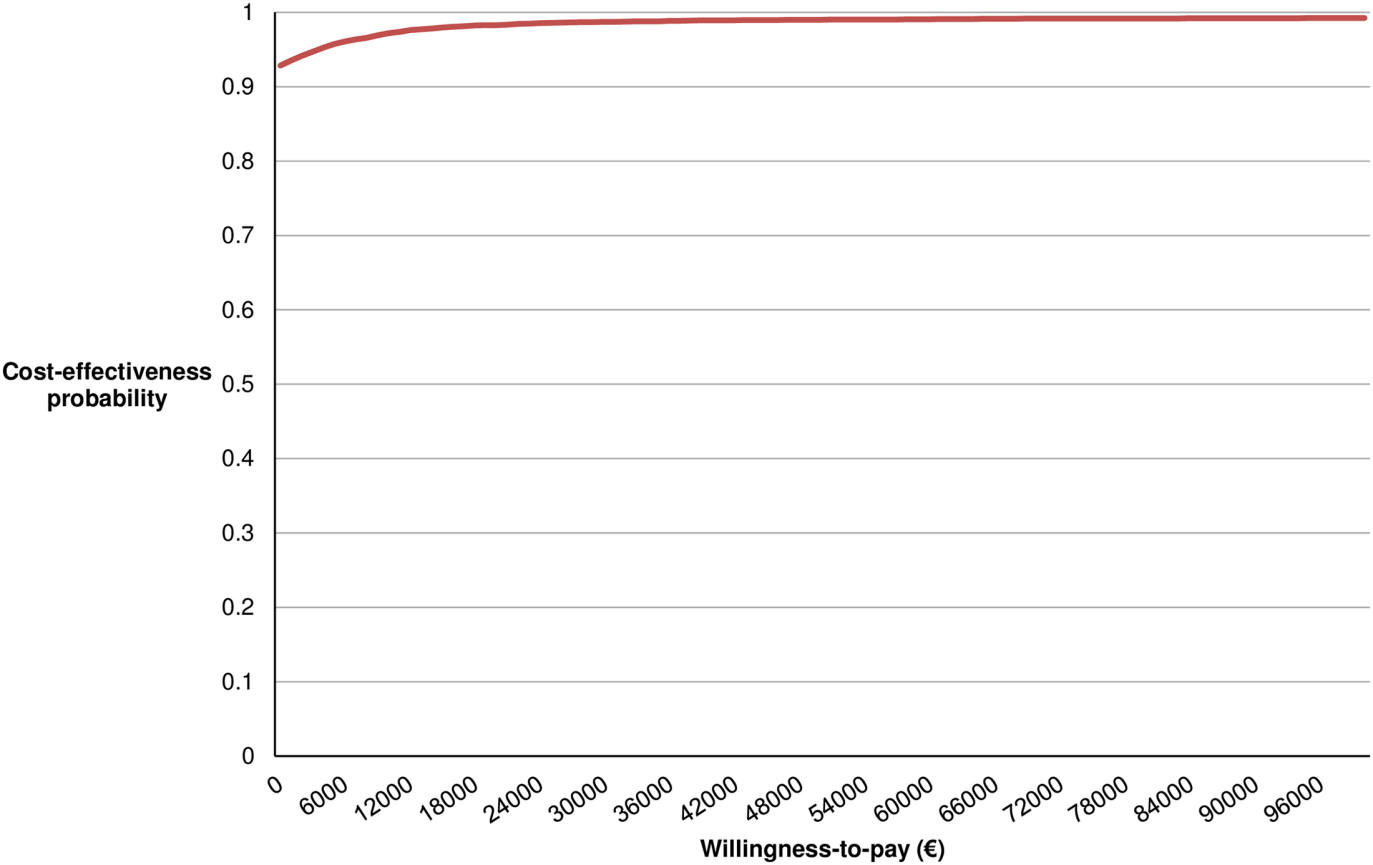
Cost-effectiveness acceptability curve.

Fig 3 shows the cost-effectiveness acceptability curve, in which the probability that the added early intervention is cost-effective in comparison with usual care is shown for a range of WTP threshold values. When society is willing to pay €20,000 per QALY, the cost-effectiveness probability of the added CBT-based early intervention is 98%.

Multiple one-way sensitivity analyses were performed in order to assess the robustness of the results. Table 3 reports the ICERs after altering different key parameters. All ICERs resulted on average in more QALYs at lower costs, indicating that the addition of the CBT-based early intervention is cost-saving on average. The confidence intervals of the reference case and sensitivity analyses ICERs indicate that there is a minimal chance that the added intervention will result in higher costs compared to usual care. However, these upper limits are far below the WTP threshold of €20,000 per QALY gained.

**Table 3:** Results of the one-way sensitivity analyses.

	ICER	95% CI	
	*Mean (€)*	*2*.*5%*	*97*.*5%*
Reference case	-23,127	-395,731	3,682
**Assumptions**			
Time horizon 1 year	-14,529	-88,655	5,276
Time horizon 10 years	-58,531	-165,535	-318
Early intervention 5% uptake rate	-23,214	-372,735	3,521
Early intervention 15% uptake rate	-23,113	-335,890	3,366
Early intervention transition from STHPD to PD 10% lower	-18,507	-246,637	4,467
Early intervention transition from STHPD to PD 10% higher	-28,351	-657,641	2,654
Uptake rate PD interventions 100%	-20,706	-264,899	4,017
Uncorrected utility values	-22,894	-314,488	3,593

## Discussion

### Main findings

This study aimed to investigate the population cost-effectiveness of adding a CBT-based early intervention for adults with STHPD to the existing health care for people with PD in the Netherlands by means of a health-economic Markov model. Our main finding was that the added early intervention for STHPD with 10% uptake yields more QALYs at lower costs on average in comparison with usual care. When a WTP-threshold of €20,000 per QALY gained is assumed, the cost-effectiveness probability of the added early intervention is 98%. Multiple one-way sensitivity analyses supported this conclusion. Therefore, adding an early intervention to the currently available interventions for PD potentially makes the care for (STH)PD in the Netherlands more cost-effective from a societal perspective.

### Comparison with other published studies in the field

Earlier, the cost-effectiveness of an early intervention in comparison with usual care was investigated alongside a trial [17]. Although this intervention was cost-effective compared to usual care, the cost-effectiveness of adding the intervention to usual care for PD at the level of the population was not assessed. Furthermore, the time horizon of a trial-based economic evaluations is relatively short. In the current study, the evidence on early interventions for STHPD was extrapolated to the population level with an extended time horizon of five years.

A comparable study in the field of mental health reported that an early intervention for patients at high risk for psychosis was also cost-saving in comparison with care as usual [37]. Our findings also suggest that offering an early intervention for STHPD has the potential to be cost-saving. Since the current evidence base on the cost-effectiveness of early interventions for STHPD is limited, more economic evaluations are needed to investigate the added value of such interventions.

### Strengths and limitations

The current study adds evidence on the cost-effectiveness of treating STHPD on the level of the population. To our knowledge, this is the first health-economic model analyzing the cost-effectiveness of adding an early intervention for STHPD to usual care for PD in the Netherlands. Available evidence regarding epidemiology, clinical effectiveness, and costs was combined to support decision-making regarding the health care system for (STH)PD. The model was designed to reflect clinical practice as closely as possible by taking into account uptake rates and adherence rates. These rates can be altered manually in the model in order to compare different scenarios.

Our study has several limitations. Ideally, model parameters are based on data reported in meta-analyses rather than single trials. Because the current evidence base on the effectiveness of early interventions for STHPD is limited, we derived effectiveness data for the early intervention from a single RCT with a sample size of 217 [16]. Although the reported preventive effect of the early intervention on the full-blown PD diagnostic status was significant, the effectiveness of the CBT-based group intervention was investigated in one trial only. Further studies assessing the effectiveness of CBT-based early interventions are needed to confirm our preliminary results.

Intervention-specific transitions were not applied for interventions for PD in the model due to a lack of available evidence. This resulted in the assumption that patients who received interventions for full-blown PD had the same chance of remission as patients who did not receive treatment. Hence, the effect of the interventions for PD might have been underestimated. However, the utility was increased as a result of receiving an intervention, which yielded QALY gains for patients who received an intervention.

Comorbid conditions were not considered in the model, which we consider as a limitation. For instance, it is known that panic is frequently comorbid with other anxiety disorders, depression, and alcohol abuse [38, 39], which could lead to higher costs and lower quality of life. The costs input parameters were corrected for comorbid mental disorders, meaning that only the costs that can be attributed to (STH)PD were considered [7, 12]. Consequently, the model does not take into account the possible effects of comorbid mental disorders on QALYs and costs. Comorbid conditions might increase societal costs and lower quality of life for individuals with (STH)PD, but it is unclear whether the effectiveness of the early intervention for STHPD is influenced by comorbid conditions.

Economic evaluations aim to identify the cost-effectiveness of interventions in order to improve efficiency in healthcare systems. It should be noted that other factors should also be taken into account in order to improve healthcare systems, such as patient preferences, feasibility, and medical ethics [40]. However, the scope of this manuscript is restricted to the cost-effectiveness perspective.

### Recommendations

Because our findings suggest that adding a CBT-based early intervention for STHPD is potentially cost-saving, policy makers should consider investing in the implementation of such interventions. However, further high-quality research is needed on the comparative long-term outcomes of early interventions as well as on costs and transition probabilities associated with STHPD and PD in order to establish the relative cost-effectiveness of early interventions for STHPD with greater certainty. Based on the study data of Batelaan et al. [17], the direct medical costs for individuals with STHPD were higher than the direct medical costs for individuals with PD. Although these findings are based on a nationally representative study sample, further studies are needed to support this finding. Finally, it should be emphasized that health-economic modeling requires empirical validation. It is thus recommended that future studies on early interventions for STHPD test the findings generated by the current model.

## Conclusions

This study showed that offering an early intervention in addition to usual care for PD in the Netherlands is potentially cost-saving. More research is needed on the comparative long-term outcomes of early interventions as well as on costs and transition probabilities associated with STHPD and PD in order to establish the relative cost-effectiveness of early interventions for STHPD with greater certainty.

## Supporting information

S1 Table CHEERS checklist—Items to include when reporting economic evaluations of health interventions. (PDF)

S2 Table Raw model input utility gain. (PDF)
