# Supplementary material for: Early intervention for subthreshold panic disorder in the Netherlands: A model-based economic evaluation from a societal perspective
Source: PLoS One. 2018 Feb 21;13(2):e0193338. doi: 10.1371/journal.pone.0193338 (PMC5821393; doi:10.1371/journal.pone.0193338)
Supplement: S2 Table — (PDF) [file pone.0193338.s002.pdf]

S2 Table

S2 table: Raw model input utility gain

Parameter	Value (mean)	SE	Source
Effect size CBT	1.142	0.075	Bandelow et al. [1]
Effect size SSRI	1.651	0.135	Bandelow et al. [1]
Effect size TCA	1.729	0.185	Bandelow et al. [1]
Effect size pill placebo	1.124	0.115	Bandelow et al. [1]
Effect size psychological placebo	0.937	0.31	Bandelow et al. [1]
Effect size pharmacotherapy+CBT	1.587	0.19	Bandelow et al. [1]
Effect size early intervention	0.791	0.17	Meulenbeek et al. [2]
**Multiplication factor for estimating utility gain (Effect size*factor)**			
Rating scale method	0.176	0.009	Sanderson et al. [3]
Time trade-off	0.109	0.011	Sanderson et al. [3]
Average used for calculations	0.260		

1. Bandelow B, Reitt M, Rover C, Michaelis S, Gorlich Y, Wedekind D. Efficacy of treatments for anxiety disorders: a meta-analysis. Int Clin Psychopharmacol. 2015;30(4):183-92. PubMed PMID: 25932596.
2. Meulenbeek P, Willemse G, Smit F, van Balkom A, Spinhoven P, Cuijpers P. Early intervention in panic: pragmatic randomised controlled trial. Br J Psychiatry. 2010;196(4):326-31. PubMed PMID: 20357312.
3. Sanderson K, Andrews G, Corry J, Lapsley H. Using the effect size to model change in preference values from descriptive health status. Qual Life Res. 2004;13(7):1255-64. PubMed PMID: 15473504.
